# Unfavorable regions in the ramachandran plot: Is it really steric hindrance? The interacting quantum atoms perspective

**DOI:** 10.1002/jcc.24904

**Published:** 2017-08-25

**Authors:** Peter I. Maxwell, Paul L. A. Popelier

**Affiliations:** ^1^ Manchester Institute of Biotechnology (MIB), 131 Princess Street, Manchester M1 7DN, Great Britain and School of Chemistry, University of Manchester, Oxford Road Manchester Great Britain M13 9PL

**Keywords:** Quantum Chemical Topology (QCT), QTAIM, rotation barrier, peptides, IQA

## Abstract

Accurate description of the intrinsic preferences of amino acids is important to consider when developing a biomolecular force field. In this study, we use a modern energy partitioning approach called Interacting Quantum Atoms to inspect the cause of the *φ* and *ψ* torsional preferences of three dipeptides (Gly, Val, and Ile). Repeating energy trends at each of the molecular, functional group, and atomic levels are observed across both (1) the three amino acids and (2) the *φ*/*ψ* scans in Ramachandran plots. At the molecular level, it is surprisingly electrostatic destabilization that causes the high‐energy regions in the Ramachandran plot, not molecular steric hindrance (related to the intra‐atomic energy). At the functional group and atomic levels, the importance of key peptide atoms (O_*i*__–1_, C_*i*_, N_*i*_, N_*i*__+1_) and some sidechain hydrogen atoms (H_γ_) are identified as responsible for the destabilization seen in the energetically disfavored Ramachandran regions. Consistently, the O_*i*__–1_ atoms are particularly important for the explanation of dipeptide intrinsic behavior, where electrostatic and steric destabilization unusually complement one another. The findings suggest that, at least for these dipeptides, it is the peptide group atoms that dominate the intrinsic behavior, more so than the sidechain atoms. © 2017 The Authors. Journal of Computational Chemistry Published by Wiley Periodicals, Inc.

## Introduction

Since the original schematic published^1^ in 1963 by Ramachandran et al., few efforts have advanced the *understanding* of the commonly denoted “forbidden” and “accepted” regions of the Ramachandran *φ*/*ψ* plot. Subsequent work by Mandel et al.^2^ allowed an advanced representation of the Ramachandran *φ*/*ψ* plot to be depicted, detailing the locations of specific hard‐sphere repulsions. Much more recently, we note that Mandel's plot is still used in undergraduate biochemistry textbooks.^3^ Hence, despite the passing of almost half a century, the hard‐sphere repulsion models have been accepted and incorporated in the development of many modern‐day molecular force fields. Perhaps regrettably, the development of these force fields focused more on successfully parameterising torsional angles rather than on understanding the quantum mechanical nature of the interactions between the atoms involved. We believe that such a greater understanding is an important step towards simplifying the parameterization task, and especially, putting it on a firmer footing. In other words, the re‐parameterization of conventional force fields typically creates new terms for experimentally observed structural effects. However, a method that directly partitions quantum mechanical information has a better chance of capturing all effects from the outset, and without extra corrections.

The conformational propensity of the 20 natural amino acids relies on three factors: intrinsic behavior, amino acid sequence, and chemical environment.^4^ Understanding the combined influence of all three aspects on a molecular system requires each factor's individual behavior to be understood first.

Addressing the first factor, that of intrinsic behavior, many studies have shown that it causes an amino acid to show preferences in *φ*/*ψ* space.^4^, ^5^, ^6^, ^7^, ^8^, ^9^, ^10^ One way of investigating intrinsic behavior is through the use of coil libraries. Coil libraries contain sequences of amino acids that form neither α‐helical or β‐sheet conformations observed in experimental X‐ray crystal structures. However, the conformations collected are always influenced by the surrounding protein structure since they are simply extracted from the initial complete protein. Hence, the individual amino acids are possibly biased by the tertiary structure of a protein. Also, they are still biased by being inside a sequence of amino acids. However, it is also known that observing isolated dipeptide structures are not representative of the amino acids behavior in oligopeptide chains.^11^ Note, as a brief aside, that the often used but actually confusing name “dipeptide” refers to a *single* amino acid, flanked by a peptide bond at both termini. Here, we must ask, at what point does an oligopeptide become a sequence, resulting in the peptide's behavior being a result of sequential effects, rather than of the intrinsic behavioral effects of its amino acids? Could it be that only the presence of intramolecular stabilization should be associated with the intrinsic behavior? Despite some investigations of single amino acids poorly replicating their behavior in larger systems, there are also many reports identifying the importance of their study.^11^, ^12^, ^13^, ^14^

The second factor is that of sequencing effects. As the name suggests, this is the effect of having a sequence of amino acids, that is, an oligopeptide. The formation of α‐helices, β‐sheets, and loops result from the specific sequence of amino acids in a chain. The α‐helices, β‐sheets, and loops are regularly occurring oligopeptide structural arrangements allowing amino acids to be packed closer together, typically through hydrogen bonding across different amino acids in the sequence. The formation of such secondary structure landmarks still rely partly on intrinsic propensity, but one could argue are dominated by the interatomic interactions formed between the sidechain and backbone atoms of neighboring amino acids in a sequence. Capturing the behavior of oligopeptide sequences still remains a non‐trivial task. A good example is given in a study by Best et al.,^15^ which recently reported the helical character induced upon tri‐, tetra‐, and penta‐Ala oligopeptides by many common force fields: GROMOS (53a6) ∼13.1%, CHARMM27 (with CMAP) ∼57.5%, AMBER03 ∼62.3%, AMBER99 ∼94.2%, and AMBER94 ∼97.6%. The experimental value each force field was striving to achieve was ∼20%. The helicity was excessive for all force fields other than GROMOS. Such is the motivation for many studies determining new or improved torsional potentials in all conventional force fields.^16^, ^17^, ^18^, ^19^, ^20^, ^21^, ^22^, ^23^

The third factor, chemical environment, is perhaps the most difficult to investigate due to the computational expense. Chemical environment behavior may be investigated through observing the influence of multiple sequences on a defined central sequence. However, chemical environment may also be an investigation of solvation effects. Both introduce many new intermolecular bonds to consider, and scale the system size dramatically.

**Figure 1:**
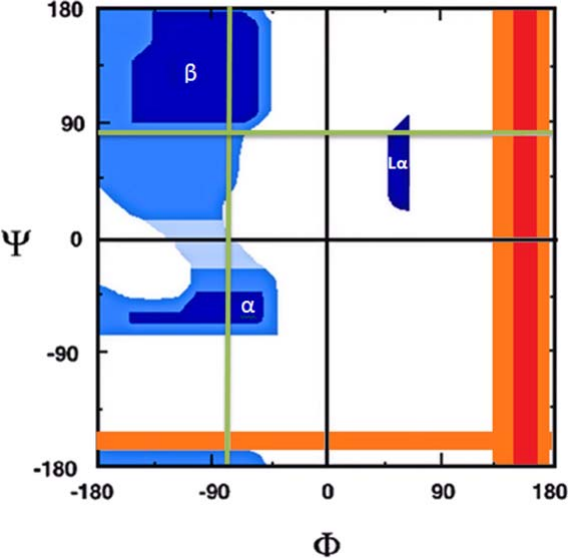
Schematic Ramachandran plot indicating the positions of β‐turns (marked as β), right‐handed helices (α), and left‐handed helices (Lα). Trajectories across the *φ* (fixed *ψ*) and *ψ* (fixed *φ*) torsional angles are indicated in green. The exact positions of the trajectories will vary slightly depending on the global minimum *φ*/*ψ* angles for each system (Gly, Ile, and Val). The crossing‐point of the green trajectories indicates the *φ*/*ψ* angles of the global minimum. The area of the Ramachandran plot is shaded according to various degrees of energetic stability: very favorable (dark blue), favorable (blue), slightly favorable (light blue), slightly unfavorable (white), unfavorable (orange) and very unfavorable (red). [Color figure can be viewed at wileyonlinelibrary.com]

Today we use quantum mechanical methods to investigate the first factor: intrinsic behavior. In order to exclusively observe the intrinsic behavior, so‐called dipeptides have been chosen as a first point of investigation. We isolate the intrinsic behavior through eliminating (i) sequencing effects by working with the single amino acid blocked (or “capped”) with an acetyl group (—COCH_3_) and an amide group (—NHCH_3_), and (ii) chemical environment through working *in vacuo*. The additional benefit of vacuum conditions, other than computational cost, is that they are important for the study of amino acids in the hydrophobic core of folded proteins typically inaccessible to solvent.^24^ Working with gas phase *ab initio* data is also in accordance with most force field development.^25^, ^26^, ^27^, ^28^, ^29^, ^30^ Our investigation aims to validate (or contrast, were appropriate) the long‐standing interpretation of the regions in the Ramachandran plot. To do this, we will use the Interacting Quantum Atoms (IQA) energy partitioning method, an approach that falls under the “umbrella” approach of Quantum Chemical Topology (QCT), a name first coined^31^ in 2003. The IQA method allows the calculation of atomic energies, which together account for the full molecular energy. The atomic energies can be classified into both intra‐ and inter‐atomic components, and also by energy type, for example, electrostatic, exchange, correlation, and so forth. Strategically chosen excursions (see Figure 1) through *φ*/*ψ* conformational space are used to obtain system conformations representing multiple regions of the Ramachandran plot. Three systems are investigated: glycine (Gly), valine (Val), and isoleucine (Ile), representing a gradual increase in the number of atoms making up the aliphatic residues, going from —NH—C_α_H_2_—CO— (in Gly) over —NH—C_α_H(C_β_H(C_γ_H_3_)_2_) —CO— (in Val) to —NH—C_α_H(C_β_ H(C_γ_H_3_)(C_γ_H_2_C_δ_H_3_)) —CO— (in Ile).

The investigation will allow us to identify the key atoms, both single and group thereof, that are responsible for both high‐ and low‐energy regions in the respective Ramachandran plots, along with any global trends that consistently appear across the three systems investigated. The existence of global trends at the atomic level will indicate transferability^32^, ^33^ within the systems, a key cornerstone of many force fields and a topic we have previously reported on for oligopeptide chain.^34^

## Methods

### Dataset generation

**Figure 2:**
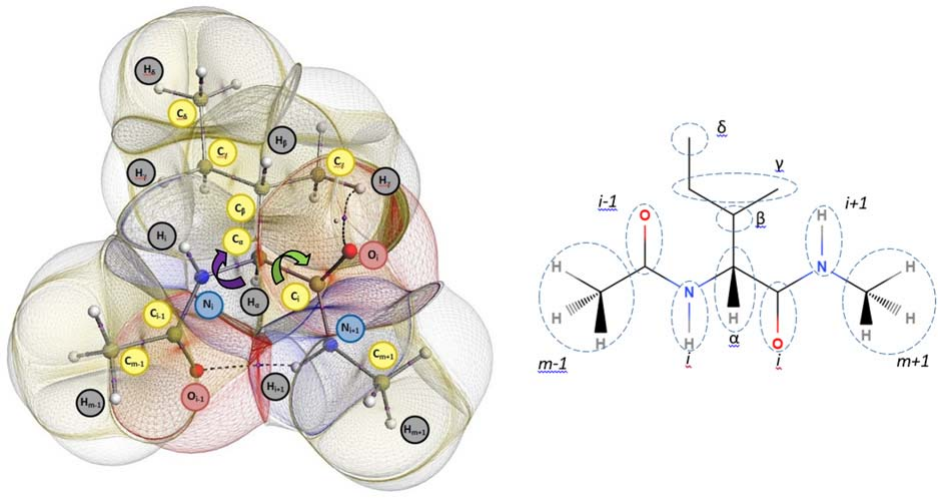
(left) Topological atoms occurring in the global energy minimum of the isoleucine dipeptide (Ile) with atom generically labelled. The dihedral angles *φ* and *ψ* are marked by a purple and green arrow, respectively. The atoms are space‐filling: they do not overlap and leave no gaps between them. Note that not all methylene or methyl hydrogen atoms are labeled in order to avoid cluttering the figure. This emblematic figure was generated by the in‐house program IRIS, which is based on previously published^36^, ^37^ algorithms. The following fragmentation will prove to make sense later in this article: CH_3_|C(=O)—N(H)|C_α_HR|C(=O) —N(H)|CH_3_, where each fragment is flanked by two vertical bars and consists of 4, 4, 15, 4, and 4 atoms, respectively, totalling 31 atoms; (right) schematic clarifying the notation in this paper (for the *i*th amino acid, which is isoleucine in this case). [Color figure can be viewed at wileyonlinelibrary.com]

The optimized geometries of the global energy minima of glycine (Gly) dipeptide, valine (Val) dipeptide, and isoleucine (Ile) dipeptide were taken from our previous work.^35^ The angle *φ* is defined as the C_*i*__–1_ ‐ N_*i*_ ‐ C_α_ ‐ C_*i*_ dihedral, and *ψ* as N_*i*_ ‐ C_α_ ‐ C_*i*_ ‐ N_*i*__+1_. Figure 2 shows these two angles and the nuclei involved in defining them. The generic notation used here will be explained in the next section. For each system in turn, the *φ* and *ψ* dihedral angles were rotated by 15° increments between −180° and +180°, resulting in 24 = [180 – (–180)]/15 geometries, additional to the global minimum. First, the *ψ* dihedral angle of the global minimum was frozen, while the *φ* angle was rotated by the increment angle over the full range (–180° ≤ *φ* ≤ +180°) using the GAUSSVIEW package. Once all 24 additional geometries for *φ* were obtained, collectively known as the *phi (φ) scan*, the procedure was repeated but now freezing the global minimum's *φ* angle and incrementing the *ψ* angle over the full range (–180° ≤ *ψ* ≤ +180°), generating the psi (*ψ*) scan. The 48 additional geometries (24 for each of the two scans in total), were then relaxed through geometry optimization but keeping both the *φ* and *ψ* dihedral angles frozen. Note that the residues were also optimized and not kept rigid relative to *φ* or *ψ*. The program GAUSSIAN09^38^ was used to perform the geometry optimization, single‐point energy calculations and printing of the wavefunction for subsequent QCT analysis, for each geometry. The optimizations and calculations were also performed at B3LYP/apc‐1 level,^39^ which is the same level of theory with which the optimized coordinates were originally obtained. In total, 6 (=2 × 3) sets of geometries were obtained, arising from two scans carried out on each of the three capped amino acids, each set consisting of 25 (=24 + 1) geometries. In summary, the overall analysis of all systems is based on 147 = 6 × 25 – 3 IQA‐partitioned wave functions, where we corrected for the fact that the three global minima are used for both *φ* and *ψ*.

After the *ab initio* calculations described above, the IQA energy partitioning calculations were performed using the AIMAll program^40^ (version 16.01.09). The non‐default settings requested in AIMAll for the IQA calculations were: the “TwoE” program for the calculation of intra‐atomic electron‐electron repulsion energies was turned off (*‐usetwoe =* 0), the target spacing between interatomic surface paths was improved from *fine* to *very fine* to ensure accurate atomic integrations (*‐iasmesh = veryfine*), and atomic IQA energies were requested (*‐encomp =* 3). The IQA energy partitioning is outlined in Section “The Interacting Quantum Atoms (IQA) Approach”, which provides only the relevant equations. In order to gauge the accuracy of AIMAll's energy partitioning, the IQA molecular energies were compared to the *ab initio* energies obtained from GAUSSIAN09. The discrepancy between this (unpartitioned) *ab initio* molecular energy and the IQA‐reconstructed molecular energy is referred to as the *IQA recovery error*. For some geometries of higher energy, the obtained IQA recovery error was considered to be too high (1 kJ mol^−1^ < *IQA recovery error* < 1.5 kJ mol^−1^). Hence, the IQA energies for these geometries were recalculated using stricter conditions for the basin outer angular quadrature (*‐boaq = skyhigh_leb* instead of the default *–boaq = auto*) in order to obtain better atomic integration accuracy. The best IQA energies, as determined by the IQA recovery error, were incorporated into the final dataset and will be reported in the Results section.

### Generic notation

Figure 2 illustrates the notation followed throughout this article. A generic notation is useful in the current study because it allows atoms that are present in all three amino acids to be identified using a single atom label and, thus, easily compared across the three amino acids. This notation is more concise than that of the unique atomic labels assigned by GAUSSVIEW, which naturally change with varying system size. The standard residue subscript labels are used, namely α, β, γ, and δ, and each is assigned to the covalently bonded atoms forming the residue. Both the carbonyl and amino groups at either side of the C_α_ are labelled with label “*i*,” which refers to the central residue as a subscript. Either side of these groups, the adjacent carbonyl and amino groups are labelled as “*i* – 1” and “*i* + 1,” respectively. So, an increasing index refers to a move towards the NHCH_3_ terminus (by convention on the right), while a decreasing index refers to moving in the opposite direction, towards the acetyl C(=O)CH_3_ terminus (on the left).

### The Interacting Quantum Atoms approach

IQA^41^ is a topological approach that sits alongside the Quantum Theory of Atoms in Molecules (QTAIM)^42^, ^43^, ^44^ and the Electron Localization Function (ELF)^45^ under the collective header of QCT.^46^, ^47^ All three share the central idea of using the gradient vector field to extract chemical information from a system. QTAIM and IQA both share the presence of topological atoms. Topological atoms, such as those seen in Figure 2 for isoleucine dipeptide, are finite‐volume three‐dimensional fragments of space representing a single atomic basin, determined by the gradient paths of a systems electron density. These atomic basins (i.e., atoms) are well‐defined even when molecules are compressed (short range van der Waals complexes), and they are space‐filling (i.e., non‐overlapping and gapless). The latter feature ensures that in an analysis of properties derived from the electron density (such as atomic energies) no part of the system is unaccounted for. This hallmark is an important advantage^48^ of QCT, particularly when applications are expanded to interactions between ligands and proteins^49^ where currently classically standardized van der Waals radii are used leaving areas of space unattributed to either the ligand or protein. The previously entitled Quantum Chemical Topological Force Field,^50^ but recently renamed to FFLUX,^51^ is a force field currently being developed with topological atoms at its heart. FFLUX features a novel design, unlike the classical designs used in other popular force fields such as AMBER and CHARMM. FFLUX maps geometrical change to a change in atomic energy through a machine learning method known as kriging.^52^ Two recent publications^50^, ^53^ describe its architecture and the process of model building in detail. FFLUX uses four primary energies to describe a molecule (or any system). The energies are obtained via the IQA energy partitioning, and include the intra‐atomic energy, the classical electrostatic energy, the exchange energy and the correlation energy. Each will be introduced in turn and described through the following equations.

IQA partitions a molecule's energy, $E_{\text{IQA}}^{\text{Mol}}$, into a sum of atomic energies, $E_{\text{IQA}}^{A}$, which in turn are composed of intra‐atomic and inter‐atomic energy components:

(1) $$E_{\text{IQA}}^{\text{Mol}}=\sum_{A}E_{\text{IQA}}^{A}=\sum_{A}E_{\text{intra}}^{A}+\frac{1}{2}\sum_{A}\sum_{B\neq A}V_{\text{inter}}^{\text{AB}}=\sum_{A}\left[E_{\text{intra}}^{A}+\frac{1}{2}\sum_{B\neq A}V_{\text{inter}}^{\text{AB}}\right]$$

where *A* and *B* represent atoms, the superscript denotes the atoms the energy is associated with and the subscript denotes the type of energy, a format that applies to all subsequent equations.

The intra‐atomic energy can be divided into its kinetic, *T*, and potential, *V*, energy contributions as follows:

(2) $$E_{\text{intra}}^{A}=T^{A}+V_{\text{ee}}^{\text{AA}}+V_{\text{en}}^{\text{AA}}$$

where *T^A^* represents the kinetic energy of atom *A*, $V_{\text{ee}}^{\text{AA}}$ is the (repulsive) potential energy between the electrons within atom *A*, and $V_{\text{ee}}^{\text{AA}}$ is the (attractive) potential energy between the electrons and nucleus of atom *A*.

Similarly, the interatomic energy can be divided into its potential energy contributions (there is no kinetic contribution this time):

(3) $$V_{\text{inter}}^{\text{AB}}=\left(V_{\text{nn}}^{\text{AB}}+V_{\text{en}}^{\text{AB}}+V_{\text{ne}}^{\text{AB}}\right)+V_{\text{ee}}^{\text{AB}}$$

where $V_{\text{en}}^{\text{AB}}$, $V_{\text{ne}}^{\text{AB}}$, and $V_{\text{ee}}^{\text{AB}}$ follow the same format as described earlier. This time the superscript and subscript ordering playing a more important role. For example, $V_{\text{en}}^{\text{AB}}$ refers to the electrons of *A* and the nucleus of *B*. Additionally, $V_{\text{nn}}^{\text{AB}}$ is the (repulsive) potential energy between the nuclei of *A* and *B*. The first three terms are bracketed to illustrate their connection to forming the “classical” electrostatic energy. To complete the electrostatic energy, $V_{\text{ee}}^{\text{AB}}$ must be expanded to:

(4) $$V_{\text{ee}}^{\text{AB}}=V_{\text{Coul}}^{\text{AB}}+V_{x}^{\text{AB}}+V_{\text{corr}}^{\text{AB}}$$

Here, “Coul” refers to the Coulombic interaction between the electrons, “x” represents the exchange energy, and “corr” the correlation energy. Now that the Coulombic energy has been separated from $V_{\text{ee}}^{\text{AB}}$, the classical electrostatic energy $V_{\text{cl}}^{\text{AB}}$ can be represented as:

(5) $$V_{\text{cl}}^{\text{AB}}=\left(V_{\text{nn}}^{\text{AB}}+V_{\text{en}}^{\text{AB}}+V_{\text{ne}}^{\text{AB}}\right)+V_{\text{Coul}}^{\text{AB}}$$

allowing the interatomic interaction energy to be rearranged to

(6) $$V_{\text{inter}}^{\text{AB}}=V_{\text{cl}}^{\text{AB}}+V_{x}^{\text{AB}}+V_{\text{corr}}^{\text{AB}}=V_{\text{cl}}^{\text{AB}}+V_{\text{xc}}^{\text{AB}}$$

This arrangement is intuitive: the classical electrostatic energy can be identified separately from the exchange and correlation energies, which together, can be thought of as the covalent contribution within an interaction.

A recent FFLUX publication^53^ introduced the use of interatomic energies designated by AA′ instead of AB. Here A′ represents every other atom in the molecular system except A. Thus, the notation AA′ denotes the interatomic energy between an atom *A* and its surrounding environment A′, such that

(7) $$\sum_{A}V_{}^{{\text{AA}}^{\prime }}≅\sum_{A}\sum_{B\neq A}V_{}^{\text{AB}}$$

The energies in eq. (7) are only approximately equivalent because they use two separate algorithms for calculation, one analytical (left term) and one numerical (right term), naturally resulting in some minor differences between the values.

In this investigation, we will also study the IQA energies at the molecular level, as well as at the atomic level and at (functional) group level (more precisely, at the level of a meaningful collection of atoms). In order to define the “molecular energies,” we observe that:

(8) $$E_{i}^{\text{Mol}}=\sum_{A}E_{i}^{A}$$

where “*i*” may be substituted for the IQA energy type of choice (i.e., *intra, IQA, cl* or *xc*). Note that for the latter two subscripts, *E* is replaced by *V* ( $E_{\text{cl}}^{A}\equiv V_{\text{cl}}^{\text{AA}\prime }$ and $E_{\text{xc}}^{A}\equiv V_{\text{xc}}^{{\text{AA}}^{\prime }}$). A particular type of molecular energy (e.g., electrostatic or exchange) is then obtained from a simple summation of the respective energy type over every atom *A*. Similarly, a particular energy of a (functional) group is obtained by energy summation over every atom belonging to the (functional) group. As a result, a hierarchical search for chemical insight can be carried out whereby first the total energy profile itself is studied ( $E_{\text{IQA}}^{\text{Mol}}$), next the various energy types at the molecular level ( $E_{i}^{\text{Mol}}$), at the (functional) group level ( $E_{i}^{G}$) (where *G* is any meaningful collection of atoms), and finally the various energy types at the atomic level ( $E_{i}^{A}$).

The IQA approach has been used to study many different chemical systems such as the interactions of Zn(II) complexes,^54^ organoselenium molecules,^55^ halogen‐trinitromethanes,^56^ halogen bonding,^57^, ^58^ and hydrogen bonding.^59^, ^60^ IQA has also been used to shed light on chemical phenomena such as steric repulsion,^61^, ^62^, ^63^ hyperconjugation,^64^ reactions,^65^ and transferability.^34^ The broad applicability of IQA, and its well‐defined and robust quantitative nature make it ideal for the current investigation. We note that IQA does not suffer from a list of conceptual and numerical problems plaguing the older and more traditional energy decomposition analysis, the many variants of which have recently been reviewed and critically discussed.^66^

The next point to highlight regards IQA's compatibility limitations, in particular the lack of affordable correlation. Until recently, IQA was incompatible at theory levels other than Hartree–Fock, full configuration interaction, configuration interaction with single and double excitations, and complete active space. This is due to perturbation theory remaining computationally very expensive even for small systems, and standard density functional theory (DFT) not providing a well‐defined second‐order reduced density matrix. However, recent developments have managed to expand IQA's application to include at least some correlation through B3LYP^67^, ^68^, ^69^ and M06–2X level DFT, and the direct correlation through coupled cluster with single and double excitations^70^, ^71^ level. In 2016, MPn‐IQA (*n* = 2, 3, or 4) also became possible.^72^ The inclusion of correlation is anticipated to have important consequences in the investigation of systems driven by dispersion energy. For further details on the expansion of IQA, the reader is directed to the respective references. Accordingly, for a more complete description of the IQA approach, the original paper of Blanco et al.^41^ should be consulted.

A final point discusses a potential concern in connection with the validity of the atomic virial theorem. Although at the root of QTAIM, this theorem is actually irrelevant for both IQA atomic energies and molecular energies, in the sense that IQA does not assume nor use the virial theorem (either atomic or molecular) in any way. We also note that self‐consistent virial scaling (SCVS) is not applicable anyway to DFT methods, such as B3LYP, so the energy partitioning in this paper could not benefit from such a correction in the first place. It is debatable whether virial‐based atomic energies are useful, in practice, even with SCVS to satisfy the molecular virial theorem. One could go as far as to state that virial‐based energies are given in AIMAll basically for historical reasons. In summary, our results are not affected by the concern raised above.

## Results and Discussion

### Preliminary analysis

The aforementioned IQA recovery error is due to the integration error, L(Ω), that accompanies each atomic integration. For our systems, the mean absolute IQA recovery errors for the *φ* scans were 0.39, 0.60, and 0.95 kJ mol^−1^ for Gly, Val, and Ile, respectively. For the *ψ* scans, they were 0.35, 0.58, and 0.57 kJ mol^−1^, respectively. With observed relative energy barriers of up to ∼64 kJ mol^−1^ and mean absolute IQA recovery errors of up to 0.95 kJ mol^−1^, the maximum percentage error of the values becomes (0.95/64) × 100 = 1.5%. We conclude that all effects seen and discussed are far above integration noise. The energy profile with the highest energy range (Ile‐*φ*) also has the highest mean absolute IQA recovery error. Larger atomic integration errors are typically observed for atoms in more complex geometries, for example, in molecules energetically far from the global energy minimum or in molecules with unusual topology.

### Analysis at molecular level

**Figure 3:**
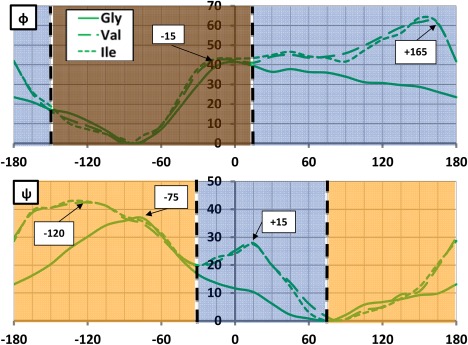
Δ $E_{\text{IQA}}^{\text{Mol}}$ (in kJ mol^−1^) scanned across *φ* (top) and *ψ* (bottom), for the Gly (solid), Val (large dashed), and Ile (small dashed) systems. All *φ* energies are relative to the optimized global minima at *φ* = −81.9°, −84.4°, and −84.1° and all *ψ* energies are relative to the optimized global minima at *ψ* =+69.8°, +83.7°, and +82.5°, respectively. The relevant energy maxima discussed in the main text are marked here for convenience. The brown area in the *φ* scan marks a region of high confluence between Gly, Val, Ile where the influence of the side chain is minimal. The navy areas in the *φ* and *ψ* scans mark regions where an extra maximum appears due to the presence of a side chain. The orange areas in the *ψ* scan mark regions where no new maxima appear but existing maxima are shifted. [Color figure can be viewed at wileyonlinelibrary.com]

**Figure 4:**
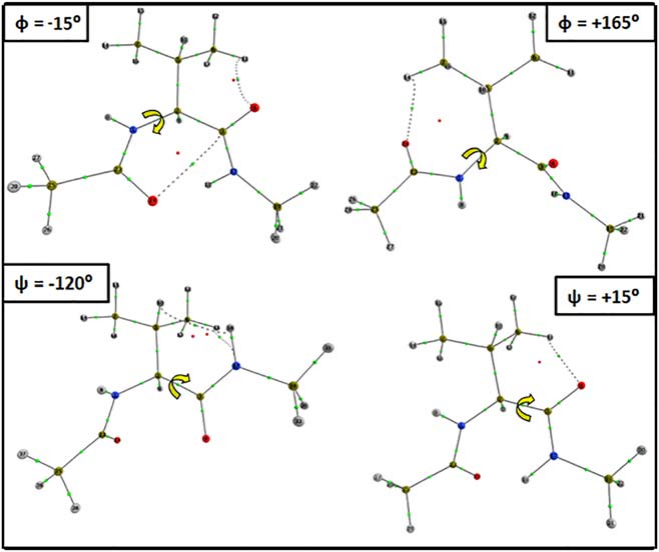
Conformations of Val at each of the maximum energy torsion angles of *φ* = −15°, *φ* = +165°, *ψ* = −120°, and *ψ* = +15° shown in Figure 3. Note that the backbone geometries also refer to the energy maxima in Ile and Gly, with the exception of the *ψ* = −75° energy maximum unique for Gly. [Color figure can be viewed at wileyonlinelibrary.com]

Figure 3 plots the $E_{\text{IQA}}^{\text{Mol}}$ energy profiles for each system, and for both the *φ* and *ψ* scans. The colored regions depict the similarity between Gly and Val/Ile energies: (1) brown indicates confluence between all three dipeptides, (2) navy indicates the appearance of an additional maximum in Val/Ile, not seen for Gly, and (3) orange indicates a change in position of a maximum seen for all three dipeptides. The first point to note is the similarity between the Val and Ile energy profiles throughout both scans. Using the Pearson correlation coefficient *r*, where *r* = 1 indicates a perfectly correlated dataset, values of *r* = 0.996 and *r* = 0.997 are obtained between the Val and Ile energy profiles within the *φ* and *ψ* scans, respectively. The striking similarity between the profile of Val and Ile is not surprising given that their side chains only differ by a methylene group. In contrast, the energy profile of Gly is less correlated to that of Val, for example, with *r* = 0.857 and *r* = 0.833 for the *φ* and *ψ* scans, respectively. However, in the *φ* scan, it is clear that the common backbone structure between Gly and Val/Ile is accountable for the molecular barrier interval of −150° ≤ *φ* ≤ +15° (brown area in Fig. 3, panel *φ*) where very similar energy profiles are observed across all three systems. Outside of this interval, we deduce that the sidechain must influence the energy profile and cause the maximal *φ* torsional barrier at +165° for Val and Ile, which is absent in Gly (navy area in Fig. 3, panel *φ*). In the *ψ* scans, the Gly and Val/Ile energy profiles are less correlated according to *r,* and indeed turn out to be more different visually. The additional local maximum at *ψ* = +15° (navy area in Fig. 3, panel *ψ*), and the translation of the maximum barrier in *ψ*, from −75° in Gly, to −120° (in Val/Ile)(orange area in Fig. 3, panel *ψ*) suggests a broader influence of the sidechain throughout the dihedral angles. In summary, the sidechain influences the energy profile more in *ψ* than in *φ*, because the former lacks the brown area of high energy profile confluence. To aid the interpretation of Figure 3, Figure 4 shows the molecular graphs of the two energy maxima in *φ* scan (–15° and +165°), and two energy maxima in the *ψ* scan (–120° and +15°).

So far, we have presented an unpartitioned perspective based on geometrical differences between Gly, Val, and Ile, allowing us to comment on the general influence of the sidechain (and its size) on the molecular energy. The current literature states that the barrier at *φ* = +165° is a result of the β‐branching on the residue causing hard‐sphere steric clashes^73^ between O_*i*__–1_ and C_β_ (at B3LYP/ANO‐L‐VDZP level), whereas the barrier at *ψ* = −120° relates to clashes^74^ between C_β_ and N_*i*__+1_. However, around *ψ* = +15°, where our earlier observations would suggest a sidechain‐related destabilization, the literature reports the region as being “sterically allowed.”^74^ This region will be investigated further later. Finally, the barrier at *φ* = −15°, which occurs in all three systems, is reportedly due to two sets of backbone clashes, one between O_*i*__–1_ and $C_{i}^{2}$, and a second^74^ between O_*i*__–1_ and N_*i*__+1_. Figure 4 is meant to help in visualising the atomic clashes mentioned above but a careful inspection may leave the impression that a more thorough atomic analysis, in the spirit of topological atoms, is needed.

To this point, we only commented on the general regions where one expects the sidechain atoms to influence (navy/orange in Fig. 3), or not (brown), the molecular energies, given the structural differences between Gly and Val/Ile. To comment further on the agreement between literature and the IQA perspective, it is necessary to partition the molecule into fragments. The IQA interpretation for the causes of the observed maxima (and minima) will now be investigated, for each system, at three partitioning levels: molecular, functional group (or collections of atoms) and atomic (i.e., single atom).

**Figure 5:**
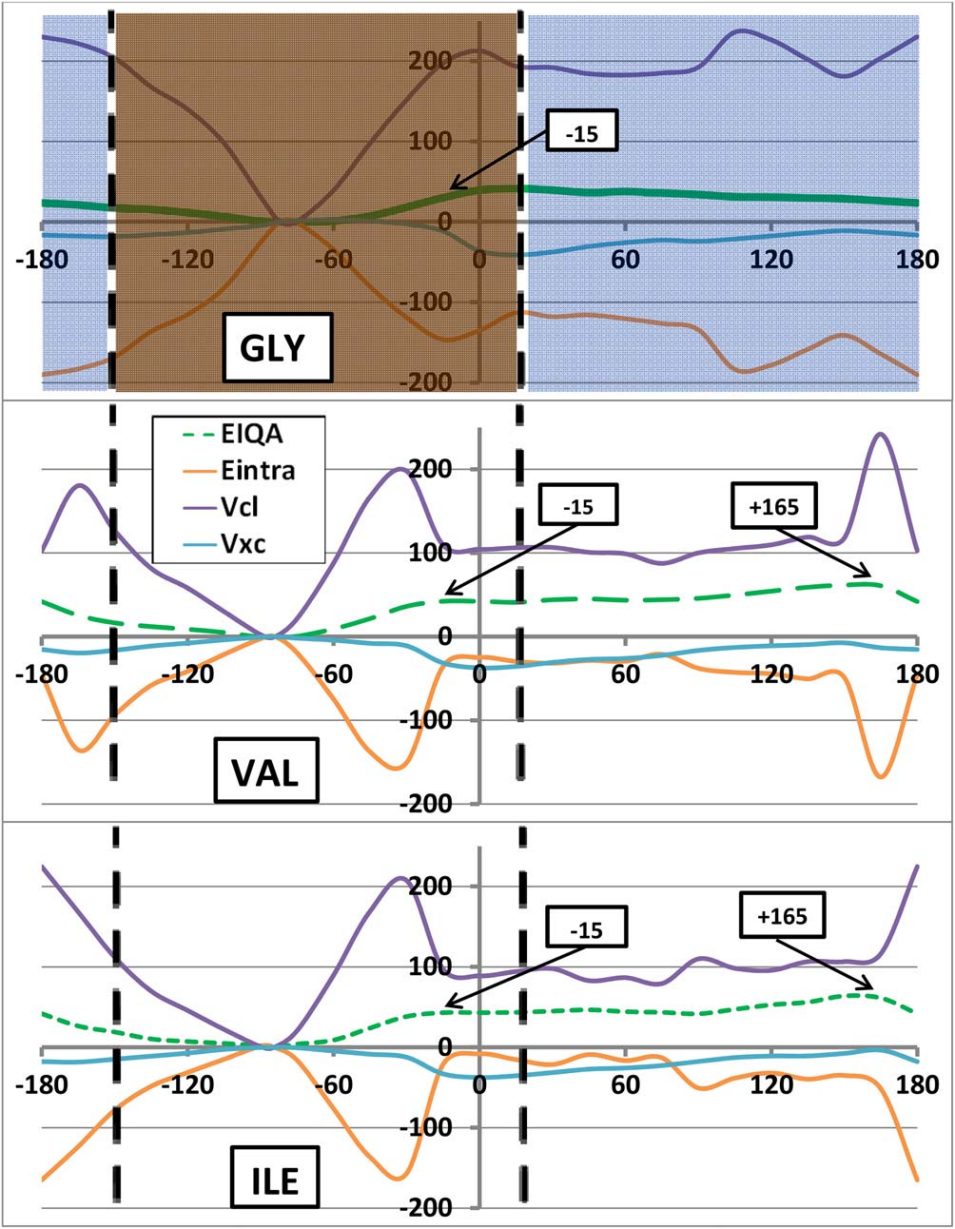
Breakdown of Δ $E_{\text{IQA}}^{\text{Mol}}$ relative energies (in kJ mol^−1^) (green) for the *φ* scan into Δ $V_{\text{cl}}^{\text{Mol}}$ (purple), Δ $E_{\text{intra}}^{\text{Mol}}$ (orange), and Δ $V_{\text{xc}}^{\text{Mol}}$ (turquoise) components for (a) Gly, (b) Val, and (c) Ile. The same convention of the green line types (solid, small dash, large dash) has been applied throughout the paper, starting in Figure 3. [Color figure can be viewed at wileyonlinelibrary.com]

**Figure 6:**
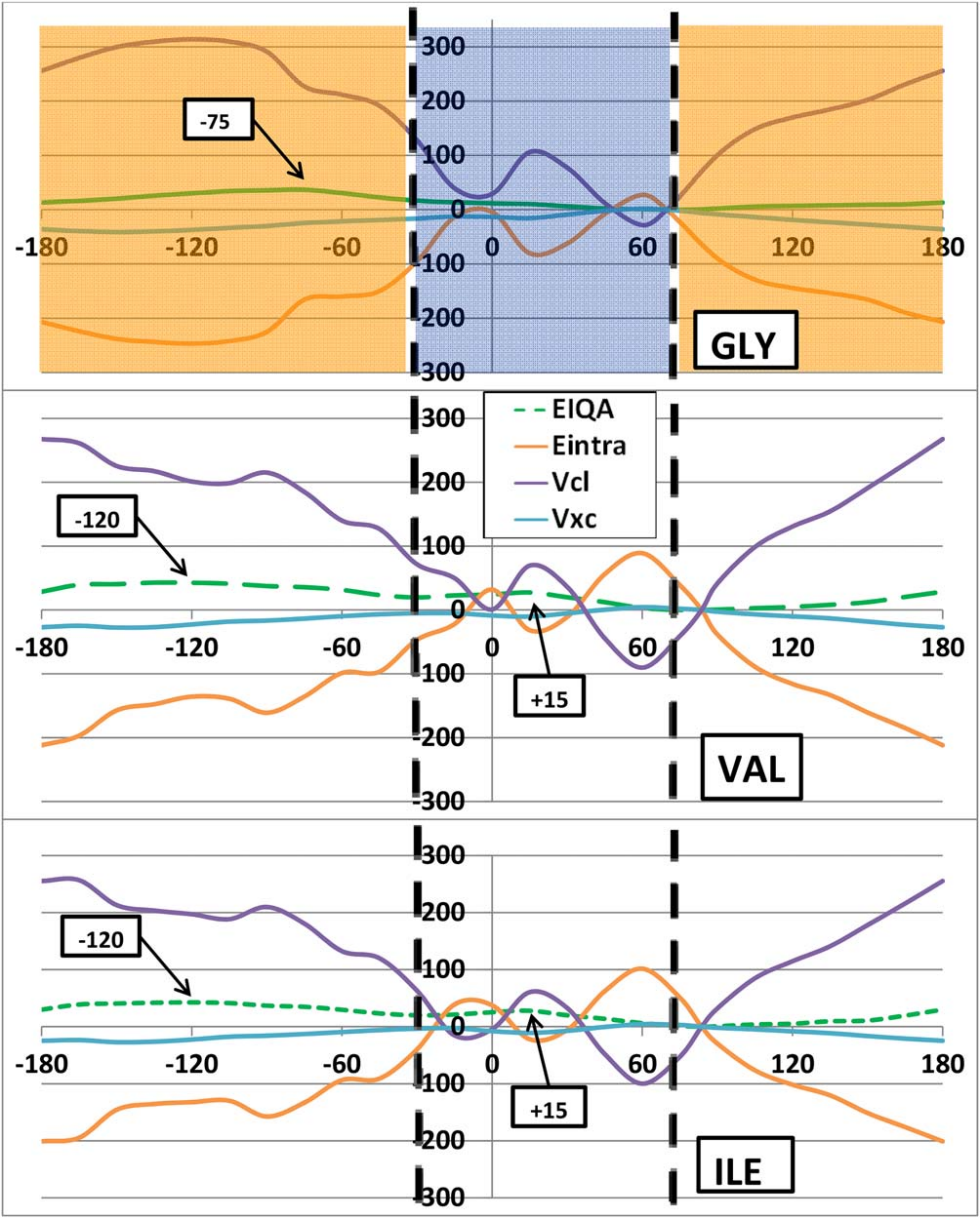
Breakdown of Δ $E_{\text{IQA}}^{\text{Mol}}$ relative energies (in kJ mol^−1^) (green) into Δ $V_{\text{cl}}^{\text{Mol}}$ (purple), Δ $E_{\text{intra}}^{\text{Mol}}$ (orange), and Δ $V_{\text{XC}}^{\text{Mol}}$ (turquoise) components for (a) Gly, (b) Val, and (c) Ile, for the *ψ* scan. The same convention of the green line types (solid, small dash, large dash) has been applied throughout the paper, starting in Figure 3. [Color figure can be viewed at wileyonlinelibrary.com]

We now analyze the overall trends of the molecular IQA energies (relative to the global minimum) illustrated for the *φ* and *ψ* scans in Figures 5 and 6, respectively. These figures show, for each of the three systems, the profile of each IQA *molecular* energy contribution to $\Delta E_{\text{IQA}}^{\text{Mol}}$, that is $\Delta V_{\text{cl}}^{\text{Mol}}$, $\Delta E_{\text{intra}}^{\text{Mol}}$, and $\Delta V_{\text{xc}}^{\text{Mol}}$ (see eq. (8)). For convenience, $\Delta E_{\text{IQA}}^{\text{Mol}}$ is plotted again for each system, repeating what was already shown in Figure 3. It is clear that the energy scale between Figure 3 and Figure 5 (or Fig. 6) differs by about an order of magnitude. This scale difference explains why the energy barriers look less pronounced at the scale of hundreds of kJ mol^−1^, which is necessary though to show the behavior of the three types of molecular energy contributions to the total molecular energy. The difference in energy scales also suggest immediately that substantial energy cancellation must take place.

Indeed, $\Delta V_{\text{cl}}^{\text{Mol}}$ and $\Delta E_{\text{intra}}^{\text{Mol}}$ broadly mirror each other, at either side of the zero energy line, and thereby more or less cancel each other. Meanwhile, $\Delta V_{\text{xc}}^{\text{Mol}}$ acts as a spectator since the absolute magnitude of its values stays of the order of tens of kJ mol^−1^ (peaking at 41 kJ mol^−1^). This also means that a curve representing $\Delta V_{\text{cl}}^{\text{Mol}}+\Delta E_{\text{intra}}^{\text{Mol}}$ (not shown in Fig. 5) would be quite similar to that of $\Delta E_{\text{IQA}}^{\text{Mol}}$. The mirroring of electrostatics by $\Delta E_{\text{intra}}^{\text{Mol}}$ is not that surprising in the light of earlier work by Martín Pendás et al*.*: charging an atom leaves behind a very important energetic fingerprint in the atomic self‐energies. *In vacuo*, the latter would increase linearly through the appropriate ionization potentials, or decrease (becoming more negative) with electron affinities (see, for instance, Ref. 75).

We learn that the cause of the energy barriers is consistent across the *φ* scan for each of the three systems: the classical electrostatic energy $\Delta V_{\text{cl}}^{\text{Mol}}$ (purple) is destabilized in the barrier regions, relative to the global minimum. Correspondingly, the height of the barriers at 0° and +165° (Val/Ile only) is dampened through stabilization of $\Delta E_{\text{intra}}^{\text{Mol}}$ (orange) and $\Delta V_{\text{xc}}^{\text{Mol}}$ (turquoise). It is important to note that $V_{\text{cl}}^{\text{Mol}}$ is always negative (attractive) across all systems and for all *φ*/*ψ* combinations. Thus, the positive *relative* (Δ) energies should be interpreted as a destabilization (i.e. a lack of stabilization) with respect to the global minimum rather than as a repulsive energy. Both $E_{\text{intra}}^{\text{Mol}}$ and $V_{\text{xc}}^{\text{Mol}}$ energies are also always negative in value, and should thus not be mistaken to be repulsive energies either.

**Figure 7:**
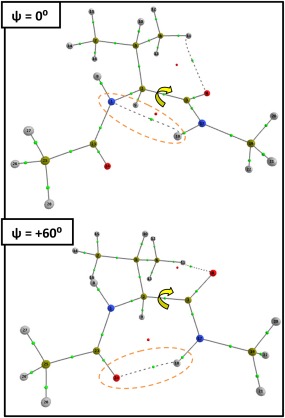
Conformations of Val illustrating the presence of (top) a 1,5 cyclic intramolecular hydrogen bond containing N_*i*_…H_*i*__+1_‐N_*i*__+1_ (orange) for *ψ* = 0° and (bottom) a 1,7 hydrogen‐bonded ring containing O_*i*__–1_…H_*i*__+1_‐N_*i*__+1_ (orange) for *ψ* = +60°. [Color figure can be viewed at wileyonlinelibrary.com]

Within the *ψ* scan, the destabilization of $\Delta V_{\text{cl}}^{\text{Mol}}$ is again the cause of the barrier within the marked orange region but both $\Delta V_{\text{cl}}^{\text{Mol}}$ and $\Delta E_{\text{intra}}^{\text{Mol}}$ fluctuate between being stabilising and destabilising in the navy region. The fluctuations result from the formation of two intramolecular hydrogen bonds causing sterically destabilising 1,5 (*ψ* = −15°) and 1,7 (*ψ* = +60°) intramolecular rings in the backbones of the dipeptides. The hydrogen bonds involve the N_*i*_…H_*i*__+1_‐N_*i*__+1_ atoms (1,5 ring) and the O_*i*__–1_…H_*i*__+1_‐N_*i*__+1_ atoms (1,7 ring), and are illustrated in Figure 7 for the Val system at conformations *ψ* = 0° (top) and *ψ* = +60° (bottom). Accompanying the destabilising intra‐atomic energy is a stabilization of the electrostatic energy within these atoms, which is expected during the formation of a hydrogen bond.

The fact that $V_{\text{cl}}^{\text{Mol}}$causes its own high‐energy regions is interesting, surprising, and perhaps even controversial. To explain why, it is necessary to refer to a recent study completed within our group where fluctuations in $E_{\text{intra}}^{}$ were observed to mimic a Buckingham‐type potential and hence significantly contribute towards steric hindrance.^76^ As a result, *the behavior of* $\Delta E_{\text{intra}}^{}$ *can be viewed as a measure of the steric hindrance*. However, this conclusion is only based on a set of observed correlations, and statistically robust fits to classical Buckingham‐type (exponential) potentials, successfully obtained for many small van der Waals complexes. Unpublished results (involving an oligopeptide intra‐atomic energy analysis) have also shown that $E_{\text{intra}}^{}$ often correlates with the atomic volume of an atom: where energy is stabilized, the atomic volume increases. Thus, the general stabilization of $\Delta E_{\text{intra}}^{\text{Mol}}$ across the energy profile should be interpreted as due to expanding atoms (i.e., relaxing) when the backbone is extending itself. Such extension occurs when *φ* or *ψ* values move towards −180° or 180°, that is, further away from the global minimum. To be more specific, of the 49 (=24 + 24 + 1) conformations (see Section 2.1) studied for each dipeptide, only four are sterically destabilized relative to the global minimum (deducible from Fig. 6, Val/Ile orange curve). At this minimum, there is an electrostatically stabilising intramolecular hydrogen bond, which explains why $\Delta E_{\text{cl}}^{\text{Mol}}$ stabilizes at nearby torsional angles (Fig. 6, Val/Ile purple curve at *ψ* = 0° and *ψ* = 60°). From this reasoning, we can initially conclude that it is generally not steric hindrance (through hard‐sphere clashes) causing the molecule to be less stable in many regions of the Ramachandran plot. Instead, the high energies are caused by a lack of electrostatic stability.

Figures 5 and 6 also show how Gly is more electrostatically destabilized than Val and Ile for many dihedral angles (both *φ* and *ψ*) on the energy profile. However, the greater electrostatic destabilization is also accompanied by a greater $\Delta E_{\text{intra}}^{\text{Mol}}$ stabilization, resulting in Val and Ile having the higher $\Delta E_{\text{IQA}}^{\text{Mol}}$, and therefore barriers, at such *φ*/*ψ* angles. Hence, IQA confirms that Gly, due to the absence of a side chain, is conformationally less restricted than other amino acids, which is expressed through greater relative stabilization via $\Delta E_{\text{intra}}^{\text{Mol}}$. These results are another example of the prominent relationship between $\Delta E_{\text{intra}}^{}$ and $\Delta V_{\text{cl}}^{}$ energies: as one becomes stabilized, the other becomes typically destabilized. This counterbalancing effect is elaborated upon in our recent work^77^ on large water clusters.

### Analysis at (functional) group level

**Figure 8:**
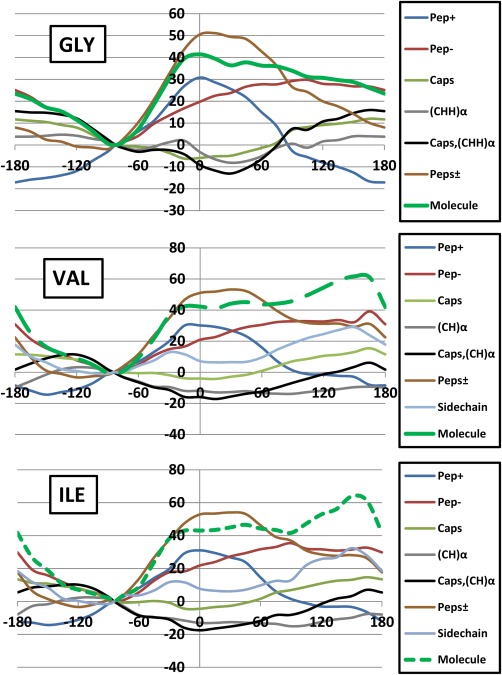
Breakdown of Δ $E_{\text{IQA}}^{\text{Mol}}$ relative energies (in kJ mol^−1^) (green) into fragment energies: Δ $E_{\text{IQA}}^{\text{Pep}+}$ (blue), Δ $E_{\text{IQA}}^{\text{Pep}\;-\;}$(red), Δ $E_{\text{IQA}}^{\text{Caps}}$ (light green), Δ $E_{\text{IQA}}^{{\left(\text{CH}\right)}_{\alpha }}$(gray), Δ $E_{\text{IQA}}^{{\text{Caps},(\text{CH})}_{\alpha }}$ (black), Δ $E_{\text{IQA}}^{\text{Peps}\pm }$ (brown), and *Δ* $E_{\text{IQA}}^{\text{sidechain}}$ (light blue) components for (a) Gly, (b) Val, and (c) Ile, for the *φ* scan. The same convention of the green line types (solid, small dash, large dash) has been applied throughout the paper, starting in Figure 3. [Color figure can be viewed at wileyonlinelibrary.com]

**Figure 9:**
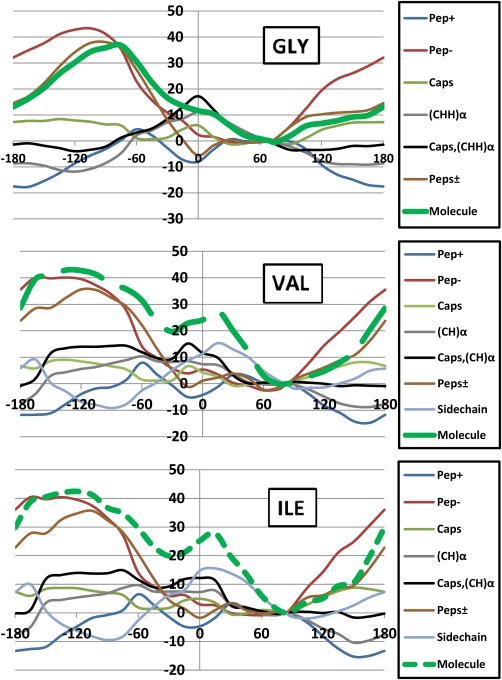
Breakdown of *Δ* $E_{\text{IQA}}^{\text{Mol}}$ relative energies (in kJ mol^−1^) (green) into fragment energies: *Δ* $E_{\text{IQA}}^{\text{Pep}+}$ (blue), *Δ* $E_{\text{IQA}}^{\text{Pep}\;-\;}$(red), *Δ* $E_{\text{IQA}}^{\text{Caps}}$ (light green), *Δ* $E_{\text{IQA}}^{{\left(\text{CH}\right)}_{\alpha }}$(gray), *Δ* $E_{\text{IQA}}^{{\text{Caps},(\text{CH})}_{\alpha }}$ (black), *Δ* $E_{\text{IQA}}^{\text{Peps}\pm }$ (brown), and *Δ* $E_{\text{IQA}}^{\text{sidechain}}$ (light blue) components for (a) Gly, (b) Val, and (c) Ile, for the *ψ* scan. The same convention of the green line types (solid, small dash, large dash) has been applied throughout the paper, starting in Figure 3. [Color figure can be viewed at wileyonlinelibrary.com]

Next we observe the functional group behavior. As mentioned in the caption of Figure 1 the following partition will prove useful: CH_3_|C(=O) —N(H)|C_α_HR|C(=O) —N(H)|CH_3_. We introduce the following notation to describe these fragments: (i) the methyl groups are combined and this collection of eight atoms is called “Caps,” (ii) the peptide group at the C‐terminus (i.e., left, involving the O_*i*__–1_ ‐ C_*i*__–1_ ‐ N_*i*_ ‐ H_*i*_) is called “Pep‐,” (iii) the peptide group at the N‐terminus (i.e., right, involving the O_*i*_ ‐ C_*i*_ ‐ N_*i*__+1_ ‐ H_*i*__+1_) is called “Pep+,” (iv) the pivotal C_α_ atom (and one H_α_ (for Ile/Val) or two H_α_ (for Gly) atoms bonded to it) is called (CH)_α_, and finally (v) the sidechain atoms (full chain for Val/Ile only, called “Sidechain”). The energies associated with the five atom groups defined above are denoted respectively: $\Delta E_{\text{IQA}}^{\text{Pep}-}$, $\Delta E_{\text{IQA}}^{\text{Pep}+\;}$, $\Delta E_{\text{IQA}}^{\text{Caps}}$, $\Delta E_{\text{IQA}}^{{\left(\text{CH}\right)}_{\alpha }}$, and $\Delta E_{\text{IQA}}^{\text{Sidechain}}$. We then sum the $\Delta E_{\text{IQA}}^{A}$ contribution of each atom forming these given groups to recover the respective group energies. We additionally sum (a) the two peptide groups denoted $\Delta E_{\text{IQA}}^{\text{Peps}\pm }$ and (b) the caps and α‐atoms into a single term denoted $\Delta E_{\text{IQA}}^{{\text{Caps},(\text{CH})}_{\alpha }}$. This grouping offers an even coarser point of view: peptides, α‐pivot and sidechains or $\Delta E_{\text{IQA}}^{\text{Peps}\pm }$, $\Delta E_{\text{IQA}}^{{\text{Caps},(\text{CH})}_{\alpha }}$, and $\Delta E_{\text{IQA}}^{\text{Sidechain}}$. Figures 8 and 9 plot the functional group analysis for the *φ* and *ψ* scans, respectively.

Figures 8 and 9 illustrate a few interesting points. First, through the newly available functional group energy profiles we can now establish a high degree of transferability, that is, a high similarity between the various energy profiles in both *φ* and *ψ* scans. This analysis allows us to dissect the consistency in the molecular energy trends seen in Figures 5 and 6. Second, the α‐atoms fluctuate very little across *φ* and *ψ* scans within ±15 kJ mol^−1^. These atoms are at the pivot point of the dihedral rotations and link the backbone to the sidechain. One would expect them to be energetically sensitive but this is not the case. Third, regions within the *φ* and *ψ* scans can be attributed to certain functional groups. As a remarkable example, we see that *the peptide groups only are responsible for the barriers in the φ scan within an interval approximately stretching from the global minimum to φ = −15*°. Outside of this interval the barrier results from both the peptide groups and the sidechain atoms. Within the *ψ* scan, again only the peptide groups are responsible for the barrier right of the global minimum (*ψ* > +75°) in all systems (allowing for discrepancies up to ∼5 kJ mol^−1^). For glycine, this remarkable match extends from *ψ* = −180° to *ψ* = −45°. Again, outside these areas, the α‐atoms and the sidechain (for Val/Ile) atoms make significant contributions towards the barrier seen at *ψ* = +15°. Collectively, the barriers at *φ* = +165° and *ψ* = +15° result from important sidechain contributions, confirming our earlier hypothesis on the rationale behind each of these maxima for Val and Ile.

The behavior of each functional group energy ( $\Delta E_{\text{intra}}^{}$, $\Delta V_{\text{cl}}^{}$, and $\Delta V_{\text{xc}}^{}$), composing the total energy of the fragment ( $\Delta E_{\text{IQA}}^{}$) may be seen in the Supporting Information in Figures S1 and S3 for *φ*, and Figures S4 to S6 for *ψ*. From these plots, it is clear that the peptide groups experience very large electrostatic and steric fluctuations across both *φ* and *ψ* scans. The remaining atom groups fluctuate less dramatically, both electrostatically and sterically. As a result, $\Delta E_{\text{intra}}^{\text{Mol}}$ and $\Delta V_{\text{cl}}^{\text{Mol}}$energy profiles are accurately (remarkably within a few kJ mol^−1^) described by profiles of the eight peptide atoms alone where: *φ* < −60° and *φ* > +150°, and −105° < *ψ* < −15° and *ψ* > +30°. However, since $\Delta E_{\text{intra}}^{\text{Pep}\pm }$ and $\Delta V_{\text{cl}}^{\text{Pep}\pm }$ cancel to a large degree, their combined contribution to $\Delta E_{\text{IQA}}^{}$ is much lower and of a comparable magnitude to that of the other group $\Delta E_{\text{intra}}^{}$ and $\Delta V_{\text{cl}}^{}$ contributions and that of $\Delta V_{\text{xc}}^{\text{Pep}\pm }$.

In summary and broadly speaking, *the molecular intra‐atomic and electrostatics are remarkably well described by the peptide atoms alone*. However, such remarkable behavior is lost when the intra‐atomic and electrostatics are added resulting in a more constrained relative energy range. In other words, the resultant cancellation and concomitant intricate interplay, leads to energy magnitudes similar to those of the remaining atomic groups ( $\Delta E_{\text{intra}}^{\text{Mol}}$ and $\Delta V_{\text{cl}}^{\text{Mol}}$) and exchange energy in general ( $\Delta V_{\text{xc}}^{}$).

Supporting Information Figure S3 shows how $\Delta V_{\text{xc}}^{}$ is dominated by only one peptide group (Pep+), which is significantly stabilized throughout each system and in *φ* scan. Supporting Information Figure S6 shows the same effect for the *ψ* scan, although not so pronounced. For clarity, Pep+ corresponds to the peptide group with H_*i*__+1_ forming an intra‐molecular hydrogen bond with the O_*i*__–1_ and N_*i*_ atoms (see Fig. 7).

### Analysis at atomic level

As a result of the group partitioning, molecular behavior has been localized to, for example, peptide atoms for certain torsional intervals. The energy profiles have also been rationalized by their electrostatic, steric, and exchange origins. Next, we take our analysis one partitioning step further and observe the energies at the atomic level. At the atomic level, we aim to isolate individual atoms causing the barriers observed within the *φ*/*ψ* scans. So far, we have learnt about the consistency of the molecular and group energy profiles across each system. At the more‐refined atomic level, we also now expect to see this consistency.

**Figure 10:**
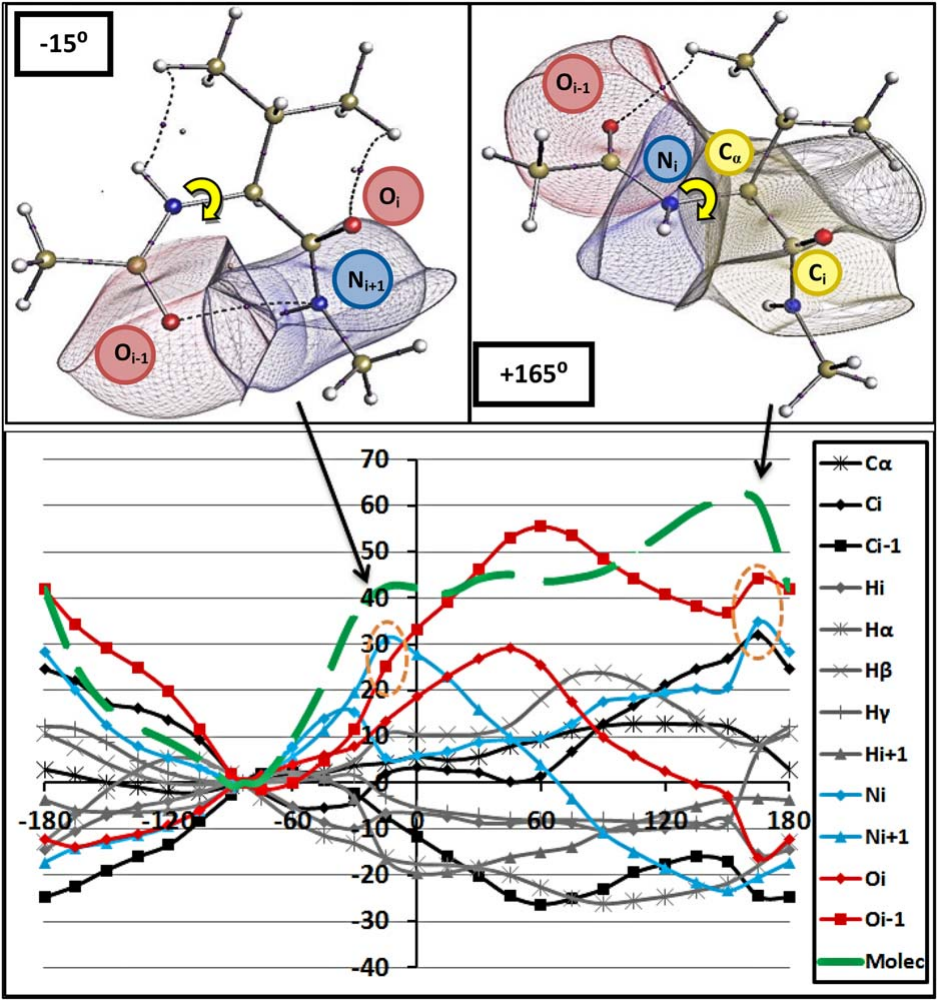
Val with *φ* = −15° (top left) and *φ* = +165° (top right) with key atomic basins depicted and the *φ* angles marked in yellow. Δ $E_{\text{IQA}}^{A}$ relative atomic energies (in kJ mol^−1^) for the *φ* scan (Bottom) for Valine with only atoms with significant energy fluctuations plotted. Traditional element colors are used for lines and symbols to distinguish element type: carbons (dark gray), hydrogens (light gray), nitrogen (blue), and oxygen (red). Symbols are indicative of the subscript of the element indicating their position in the molecule. Δ $E_{\text{IQA}}^{\text{Mol}}$ energy is given in green. Orange circles depict most destabilized atoms in each barrier region. The same convention of the green line types (solid, small dash, large dash) has been applied throughout the paper, starting in Figure 3. [Color figure can be viewed at wileyonlinelibrary.com]

**Figure 11:**
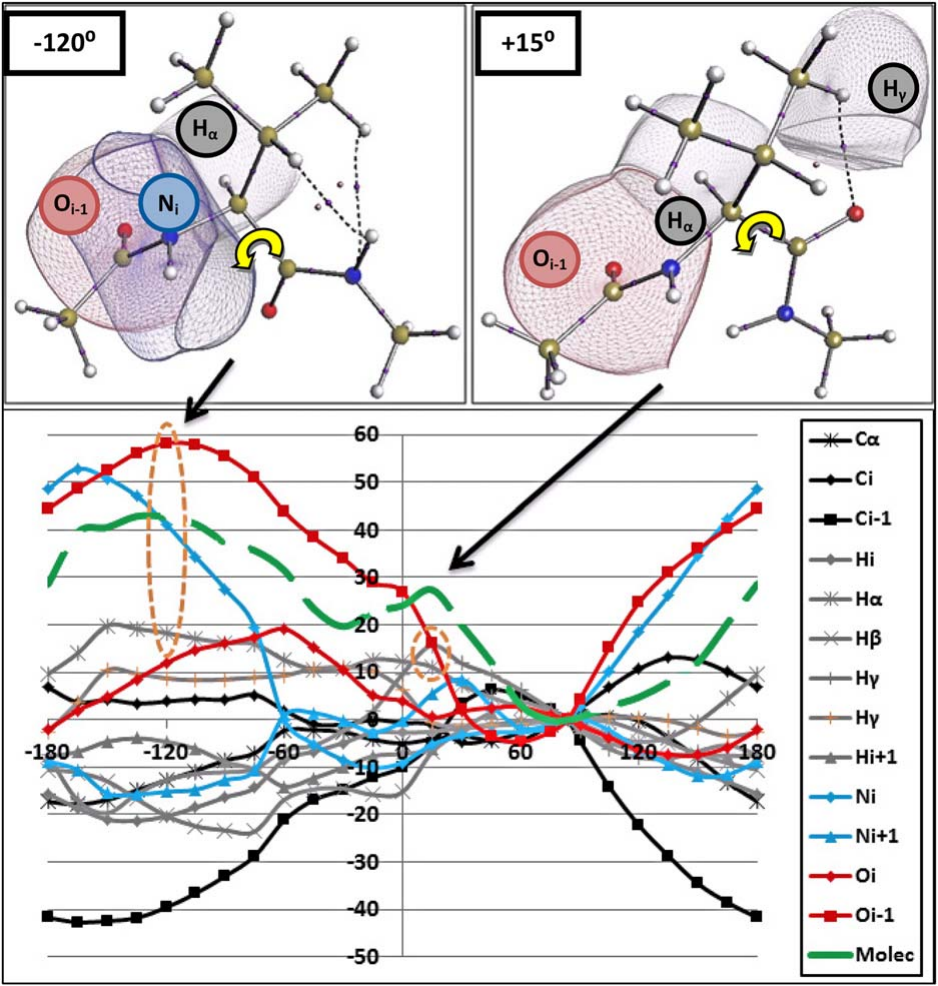
Val with *ψ* = −120° (top left) and *ψ* = +15° (top right) with key atomic basins depicted and the *ψ* angles marked in yellow. Δ $E_{\text{IQA}}^{A}$ relative atomic energies (in kJ mol^−1^) for the *ψ* scan (Bottom) for Valine with only atoms with significant energy fluctuations plotted. Traditional element colors are used for lines and symbols to distinguish element type: carbons (dark gray), hydrogens (light gray), nitrogen (blue), and oxygen (red). Symbols are indicative of the subscript of the element indicating their position in the molecule. Δ $E_{\text{IQA}}^{\text{Mol}}$ energy is given in green. Orange circles depict most destabilized atoms in each barrier region. The same convention of the green line types (solid, small dash, large dash) has been applied throughout the paper, starting in Figure 3. [Color figure can be viewed at wileyonlinelibrary.com]

Figures 10 and 11 plot the $\Delta E_{\text{IQA}}^{A}$ energy profiles for key atoms in the Val *φ* and *ψ* scans, respectively. Supporting Information Figures S7 and S8 plot the same type of information for the remaining two systems: Gly and Ile. Here, we only report the Val results because it is clear that the atomic trends are very similar throughout each system, within each scan. To clarify, where backbone atomic destabilization is observed within Gly, is it equally present within Val. In addition, by comparing Figures 10 and 11 to Supporting Information Figures S7 and S8, it is clear the Val and Ile plots are almost identical for every torsional angle. Hence, we focus on the general trends using Val as the example.

In Figures 10 and 11, we plot only the key atoms with energy fluctuations greater than ±10 kJ mol^−1^. Many sidechain and methyl‐cap atoms fell below this threshold and are hence not included in these figures. In fact, very few sidechain atoms fluctuate with any significant energy deviations, except for two H_ϒ_ atoms. In addition, the C_α_ atoms also fluctuate very little (< ±8 kJ mol^−1^) but are included in the plot to demonstrate this key point. In the group analysis, we already established a lack of energy fluctuation for C_α_ atoms but *we reiterate this surprising result considering C_α_'s key bridging role*. The O_*i*__–1_ atoms are the most perturbed atoms across all six torsional scans, indicating their importance to the overall molecular stability. In contrast to the destabilising behavior of the O_*i*__–1_ atoms, the vicinal C_*i*__–1_ atoms significantly stabilize throughout. Within the *φ* scan of Figure 10, we also see N_i_ becoming the most destabilized atom when −90° < *φ* < 0° and. In the *ψ* scan of Figure 11, N_*i*_ starts to match O_i‐1_ in terms of destabilization magnitude in the vicinity of the *ψ* = −120° barrier. We learn that the O_*i*__–1_ and N_*i*_ atoms dominate the destabilization within each system, across both scans. Many of the remaining atoms fluctuate with some preference towards stabilization or destabilization, or oscillate around the zero‐energy line (given by the global minimum).

Figures 10 and 11 also plot the atomic basins for the most destabilising atoms present at the barriers at *φ* = −15°, *φ*= +165°, and *ψ* = −120°, *ψ* = +15°, respectively. The literature reasoning behind each barrier will now be further compared with the IQA‐based reasoning. Both Mandel et al.^2^ and Ho et al.^74^ state that the clash between O_*i*__–1_ and N_*i*__+1_ contributes to the barrier at *φ* = −15°. The $\Delta E_{\text{IQA}}^{A}$ analysis shown in Figure 10 confirms this because the largest destabilising (i.e., positive energy) contributors to this barrier are indeed O_*i*__–1_ and N_*i*__+1_. However, Mandel et al.^2^ quotes the clash between O_*i*__–1_ and C_*i*_ as an extra contributor to this barrier, which we *cannot* confirm because the positive $\Delta E_{\text{IQA}}^{A}$ for C_i_ is an order of magnitude smaller than that of N_*i*__+1_.

We now analyze the barrier at *φ* = +165° in a similar way. Ho et al.^74^ suggest that a clash between O_*i*__–1_ and C_β_ causes this barrier. Our analysis confirms that O_*i*__–1_ is indeed a major factor of destabilization (large positive $\Delta E_{\text{IQA}}^{A}$value) but C_β_ is not at all (in fact, because it is always smaller than 4 kJ mol^−1^ is not even shown in Fig. 10). However, if our analysis is forced to point out destabilising atoms from the side chain then one H_β_ and one H_ϒ_ atom emerge. Much more significant destabilization originates from N_*i*_ and C_*i*_. We are now in a position to refine our earlier observation in the molecular energy analysis (see Section “Analysis at molecular level”). Although the sidechain causes the *φ* = +165° barrier, it results from three peptide atoms (O_*i*__–1_, C_*i*_, and N_*i*_) being destabilized alongside two sidechain hydrogen atoms but not sidechain carbons.

Within the *ψ* scans, we do not see the reported^74^ clash between N_*i*__+1_ and C_β_ when *ψ* = −120°. Instead, we observe that N_i_ and O_i‐1_ are most destabilized alongside H_α_ (see Fig. 11). Moreover, the suggested N_*i*__+1_ is actually stabilising at *ψ* = −120°, according to our findings. For the *ψ* = +15° barrier, which is known to be sterically allowed but without specific clashing atoms identified, we discover that the sidechain (within Val/Ile) is destabilized through H_ϒ_. Indeed, in Figure 11 we see a clear peak at *ψ* = +15° for H_ϒ_. In addition to H_ϒ_ being destabilized, H_α_ and O_*i*__–1_ are also destabilising when *ψ* = +15°.

Overall, some of our atomic interpretations of energy barriers are quite different to those in previous literature. However, our energies are more complete than those represented by, for example, the hard‐sphere model, which only considers the steric‐like behavior of an atom. To better understand the nature of the destabilization of each atom, it is necessary to observe the causal energies ( $\Delta E_{\text{intra}}^{A}$, $\Delta V_{\text{cl}}^{\text{AA}\prime }$, and $\Delta V_{\text{xc}}^{\text{AA}\prime }$) composing $\Delta E_{\text{IQA}}^{A}$. The Supporting Information reports each of these three atomic energy profiles in Figures S9 to S11 for the *φ* scan, and again in Supporting Information Figures S12 to S14 for the *ψ* scan. Collectively, Supporting Information Figures S9 to S14 allow us to identify the source of destabilization for every atom known to be significantly destabilized (through $\Delta E_{\text{IQA}}^{A}$) at the barrier peaks. The results are summarized in Table 1.

**Table 1:** Summary of depicted atomic basins and their origin of destabilization.

Barrier	Atom	Origin of destabilization
*φ* = −15°	O*_*i*_*_–1_ N*_*i*_*_+1_	Steric Electrostatic
*φ* = +165°	O*_*i*_*_–1_ N*_*i*_* C*_*i*_* H_β_ and H_ϒ_ a	Steric and Electrostatic Electrostatic and Exchange Steric and Electrostatic Steric
*Ψ* = −120°	O*_*i*_*_–1_ N*_*i*_*	Steric and Electrostatic Electrostatic
*Ψ* = +15°	O*_*i*_*_–1_ H_α_ H_ϒ_	Electrostatic and Exchange Steric and Exchange Steric

a Their atomic basins are not drawn in Figure 10 but collectively contribute around the barrier.

We note an unusual result for two cases: O_i‐1_ (*φ* = +165° and *Ψ* = −120°) and C_i_ (*φ* = +165°), where we observe the anomalous combination of both significantly destabilising steric (intra‐atomic) and destabilising electrostatic energies. For O_*i*__–1_ in particular, the anomalous lack of cancellation causes the atom to be the most destabilized (through $\Delta E_{\text{IQA}}^{A}$) atom across each of all six torsional scans.

Energy profile consistency has been identified within both the molecular energy analysis and the (functional) group analysis. When a system is partitioned, consistency of energy trends is commonly known as “transferability,” which is a key topic in force field design. If atomic energies (group or single atoms) are identified as being consistent across systems, then such atomic energies are said to be transferable. Categorising such transferable atoms should become a significant topic within computational chemistry itself.

Finally, we comment further on the transferable energy trends. Some weak trends occur in the backbone atoms when comparing Gly with Val/Ile but they are strengthened when comparing Val and Ile. Within the atomic analysis, Gly and Val atomic energies are similar to within 8 kJ mol^−1^ (while −165° < *φ* < +15°) and within 9 kJ mol^−1^ (across all *ψ* angles). The Val and Ile atomic energies are even closer, within 5 kJ mol^−1^ (while −165° < *φ* < +165°) and within 3 kJ mol^−1^ (when *ψ* < 0° or *ψ* > 0°). The minimal energy discrepancies across Gly, Val, and Ile corroborate fragment transferability, which force field developers need in their atom typing. The results presented also support some of our other work^34^ on IQA and transferability.

## Conclusions

In this study, three dipeptides (Gly, Val, and Ile) were investigated to gain a better understanding of the intrinsic behavior of amino acids at three successive levels of detail: molecular, (functional) group, and atomic. The topological energy partitioning method called IQA provided four types of energy to achieve this goal: intra‐atomic (self) energy ( $E_{\text{intra}}^{}$), electrostatic energy ( $V_{\text{cl}}^{}$), exchange(‐correlation) energy ( $V_{\text{xc}}^{}$), and the sum of all three ( $E_{\text{IQA}}^{}$). We determined the causes of the high‐energy regions at relevant combinations of *φ*/*ψ* in the Ramachandran plots.

At molecular level, a destabilization of the electrostatic energy is the cause of the barrier regions across both *φ* and *ψ* scans, and across each dipeptide system. However, each electrostatic barrier is dampened by counter‐stabilization from $\Delta E_{\text{intra}}^{\text{Mol}}$ and $\Delta V_{\text{xc}}^{\text{Mol}}$. Electrostatics dictating the barriers is an unexpected conclusion given the prevailing view that steric hindrance can explain the Ramachandran regions.

At atom‐group level, the peptide groups are consistently the cause of the barriers at *φ* = −15° and *ψ* = −120°, with the barriers at *φ* = +165° and at *ψ* = +15° arising as the result of both the peptides and sidechain groups becoming destabilized, cooperatively.

At atomic level (*A*), the aforementioned group trends were reflected in destabilized $\Delta E_{\text{IQA}}^{A}$ energies for key peptide atoms (O_*i*__–1_, C_*i*_, N_*i*_, and N_*i*__+1_) and some sidechain hydrogen atoms (H_β_ and H_γ_).

The origin of the atomic destabilization was also clarified through the analysis of the $\Delta E_{\text{intra}}^{A}$, $\Delta V_{\text{cl}}^{\text{AA}\prime }$, and $\Delta V_{\text{xc}}^{\text{AA}\prime }$ energies (A′ is the atomic environment of *A*), confirming some steric destabilization within the O_*i*__–1_, C_*i*_, H_α_ and sidechain H_γ_ and H_β_ atoms at barrier peaks. Surprisingly and interestingly, the energies of the sidechain carbon atoms (C_β_, C_ϒ_, C_δ_), and more importantly C_α_, remained relatively unperturbed throughout.

Finally, some very promising results regarding transferability were observed where absolute values of atomic energies are smaller than 9 kJ mol^−1^ between Gly and Val/Ile, and smaller than 5 kJ mol^−1^ between Val and Ile, for the majority of torsional angles across both *φ*/*ψ* scans.

## Supporting information

Supporting Information
