# Supplementary material for: Unfavorable regions in the ramachandran plot: Is it really steric hindrance? The interacting quantum atoms perspective
Source: J Comput Chem. 2017 Aug 25;38(29):2459–74. doi: 10.1002/jcc.24904 (PMC5659141; doi:10.1002/jcc.24904)
Supplement: Supplementary file 1 — Supporting Information [file JCC-38-2459-s001.docx]

**Supporting Information**

**Unfavourable Regions in the Ramachandran plot: Is it Really Steric Hindrance? The Interacting Quantum Atoms (IQA) Perspective**

P. I. Maxwell and P. L. A. Popelier

Manchester Institute of Biotechnology (MIB), 131 Princess Street, Manchester M1 7DN, Great Britain and School of Chemistry, University of Manchester, Oxford Road, Manchester M13 9PL, Great Britain.

**Figures S1 to S6.** Breakdown of Δ$E_{\text{intra}}^{\text{Mol}}$, Δ$V_{\text{cl}}^{\text{Mol}}$ and Δ$V_{\text{xc}}^{\text{Mol}}$ relative energies (orange) into fragment energies: Δ$E_{\text{IQA}}^{\text{Pep+}}$ (blue), Δ$E_{\text{IQA}}^{\text{Pep - }}$(red), Δ$E_{\text{IQA}}^{\text{Caps}}$ (light green), Δ$E_{\text{IQA}}^{\text{(CH)}_{\alpha}}$(grey), Δ$E_{\text{IQA}}^{\text{Caps,(CH)}_{\text{α}}}$ (black), Δ$E_{\text{IQA}}^{\text{Peps}\pm}$ (brown) and Δ$E_{\text{IQA}}^{\text{sidechain}}$ (light blue) components for (a) Gly, (b) Val and (c) Ile, for the φ (S1 to S3) and ψ (S4 to S6) scans.

**Figures S7 and S8.** Relative Δ$E_{\text{IQA}}^{\text{A}}$ total atomic energies for the scan across φ (S7) and ψ (S8) for Gly (top) and Ile (bottom). Only the highest energetically fluctuating atoms are plotted. Element colours are used for plot lines to distinguish element type: carbons (dark grey), hydrogens (light grey), nitrogen (blue) and oxygen (red). The molecular Δ$E_{\text{IQA}}^{\text{A}}$ energy is given by a single thick dark orange line. Generic atom labels are used in the legend and correspond to Figure 2 in the main text.

**Figures S9 to S14.** Relative Δ$E_{\text{intra}}^{\text{A}}$, Δ$V_{\text{cl}}^{\text{A}}$ and Δ$V_{\text{xc}}^{\text{A}}$ total atomic energies for the scan across φ (S9 to S11) and ψ (S12 to S14) for Gly (top), Val (middle) and Ile (bottom). Only the highest energetically fluctuating atoms are plotted. Element colours are used for plot lines to distinguish element type: carbons (dark grey), hydrogens (light grey), nitrogen (blue) and oxygen (red). The molecular Δ$E_{\text{intra}}^{\text{A}}$ , Δ$V_{\text{cl}}^{\text{A}}$ and Δ$V_{\text{xc}}^{\text{A}}$ energies are given by a single thick dark orange, purple or turquoise line, respectively. Generic atom labels are used in the legend and correspond to Figure 2 in the main text.

**Figure S15.** Alteration of Figure 5c of the main manuscript, with the addition of a further summed Δ$V_{\text{cl}}^{\text{Mol}}$ (purple) + Δ$E_{\text{intra}}^{\text{Mol}}$ (orange) energy (represented as brown). Energies are in kJmol^-1^ for the Ile ϕ scan.

**Figure S1.** Breakdown of Δ$E_{\text{intra}}^{\text{Mol}}$ relative energies (orange) into fragment energies: Δ$E_{\text{IQA}}^{\text{Pep+}}$ (blue), Δ$E_{\text{IQA}}^{\text{Pep - }}$(red), Δ$E_{\text{IQA}}^{\text{Caps}}$ (light green), Δ$E_{\text{IQA}}^{\text{(CH)}_{\alpha}}$(grey), Δ$E_{\text{IQA}}^{\text{Caps,(CH)}_{\text{α}}}$ (black), Δ$E_{\text{IQA}}^{\text{Peps}\pm}$ (brown) and Δ$E_{\text{IQA}}^{\text{sidechain}}$ (light blue) components for (a) Gly, (b) Val and (c) Ile, for the φ scan.

**Figure S2.** Breakdown of Δ$V_{\text{cl}}^{\text{Mol}}$ relative energies (purple) into fragment energies: Δ$E_{\text{IQA}}^{\text{Pep+}}$ (blue), Δ$E_{\text{IQA}}^{\text{Pep - }}$(red), Δ$E_{\text{IQA}}^{\text{Caps}}$ (light green), Δ$E_{\text{IQA}}^{\text{(CH)}_{\alpha}}$(grey), Δ$E_{\text{IQA}}^{\text{Caps,(CH)}_{\text{α}}}$ (black), Δ$E_{\text{IQA}}^{\text{Peps}\pm}$ (brown) and Δ$E_{\text{IQA}}^{\text{sidechain}}$ (light blue) components for (a) Gly, (b) Val and (c) Ile, for the φ scan.

**Figure S3.** Breakdown of Δ$V_{\text{xc}}^{\text{Mol}}$ relative energies (turquoise) into fragment energies: Δ$E_{\text{IQA}}^{\text{Pep+}}$ (blue), Δ$E_{\text{IQA}}^{\text{Pep - }}$(red), Δ$E_{\text{IQA}}^{\text{Caps}}$ (light green), Δ$E_{\text{IQA}}^{\text{(CH)}_{\alpha}}$(grey), Δ$E_{\text{IQA}}^{\text{Caps,(CH)}_{\text{α}}}$ (black), Δ$E_{\text{IQA}}^{\text{Peps}\pm}$ (brown) and Δ$E_{\text{IQA}}^{\text{sidechain}}$ (light blue) components for (a) Gly, (b) Val and (c) Ile, for the φ scan.

**Figure S4.** Breakdown of Δ$E_{\text{intra}}^{\text{Mol}}$ relative energies (orange) into fragment energies: Δ$E_{\text{IQA}}^{\text{Pep+}}$ (blue), Δ$E_{\text{IQA}}^{\text{Pep - }}$(red), Δ$E_{\text{IQA}}^{\text{Caps}}$ (light green), Δ$E_{\text{IQA}}^{\text{(CH)}_{\alpha}}$(grey), Δ$E_{\text{IQA}}^{\text{Caps,(CH)}_{\text{α}}}$ (black), Δ$E_{\text{IQA}}^{\text{Peps}\pm}$ (brown) and Δ$E_{\text{IQA}}^{\text{sidechain}}$ (light blue) components for (a) Gly, (b) Val and (c) Ile, for the ψ scan.

**Figure S5.** Breakdown of Δ$V_{\text{cl}}^{\text{Mol}}$ relative energies (purple) into fragment energies: Δ$E_{\text{IQA}}^{\text{Pep+}}$ (blue), Δ$E_{\text{IQA}}^{\text{Pep - }}$(red), Δ$E_{\text{IQA}}^{\text{Caps}}$ (light green), Δ$E_{\text{IQA}}^{\text{(CH)}_{\alpha}}$(grey), Δ$E_{\text{IQA}}^{\text{Caps,(CH)}_{\text{α}}}$ (black), Δ$E_{\text{IQA}}^{\text{Peps}\pm}$ (brown) and Δ$E_{\text{IQA}}^{\text{sidechain}}$ (light blue) components for (a) Gly, (b) Val and (c) Ile, for the ψ scan.

**Figure S6.** Breakdown of Δ$V_{\text{xc}}^{\text{Mol}}$ relative energies (turquoise) into fragment energies: Δ$E_{\text{IQA}}^{\text{Pep+}}$ (blue), Δ$E_{\text{IQA}}^{\text{Pep - }}$(red), Δ$E_{\text{IQA}}^{\text{Caps}}$ (light green), Δ$E_{\text{IQA}}^{\text{(CH)}_{\alpha}}$(grey), Δ$E_{\text{IQA}}^{\text{Caps,(CH)}_{\text{α}}}$ (black), Δ$E_{\text{IQA}}^{\text{Peps}\pm}$ (brown) and Δ$E_{\text{IQA}}^{\text{sidechain}}$ (light blue) components for (a) Gly, (b) Val and (c) Ile, for the ψ scan.

**Figure S7.** Relative Δ$E_{\text{IQA}}^{\text{A}}$ total atomic energies for the scan across φ for Gly (top) and Ile (bottom). Only the highest energetically fluctuating atoms are plotted. Element colours are used for plot lines to distinguish element type: carbons (dark grey), hydrogens (light grey), nitrogen (blue) and oxygen (red). The molecular Δ$E_{\text{IQA}}^{\text{A}}$ energy is given by a single thick dark green line. Generic atom labels are used in the legend and correspond to Figure 2 in the main text.

**Figure S8.** Relative Δ$E_{\text{IQA}}^{\text{A}}$ total atomic energies for the scan across ψ for Gly (top) and Ile (bottom). Only the highest energetically fluctuating atoms are plotted. Element colours are used for plot lines to distinguish element type: carbons (dark grey), hydrogens (light grey), nitrogen (blue) and oxygen (red). The molecular Δ$E_{\text{IQA}}^{\text{A}}$ energy is given by a single thick dark green line. Generic atom labels are used in the legend and correspond to Figure 2 in the main text.

**Figure S9.** Relative Δ$E_{\text{intra}}^{\text{A}}$ total atomic energies for the scan across φ for Gly (top), Val (middle) and Ile (bottom). Only the highest energetically fluctuating atoms are plotted. Element colours are used for plot lines to distinguish element type: carbons (dark grey), hydrogens (light grey), nitrogen (blue) and oxygen (red). The molecular Δ$E_{\text{intra}}^{\text{A}}$ energy is given by a single thick dark orange line. Generic atom labels are used in the legend and correspond to Figure 2 in the main text.

**Figure S10.** Relative Δ$V_{\text{cl}}^{\text{AA'}}$ total atomic energies for the scan across φ for Gly (top), Val (middle) and Ile (bottom). Only the highest energetically fluctuating atoms are plotted. Element colours are used for plot lines to distinguish element type: carbons (dark grey), hydrogens (light grey), nitrogen (blue) and oxygen (red). The molecular Δ$V_{\text{cl}}^{\text{AA'}}$ energy is given by a single thick dark purple line. Generic atom labels are used in the legend and correspond to Figure 2 in the main text.

**Figure S11.** Relative Δ$V_{\text{xc}}^{\text{AA'}}$^’^ total atomic energies for the scan across ψ for Gly (top), Val (middle) and Ile (bottom). Only the highest energetically fluctuating atoms are plotted. Element colours are used for plot lines to distinguish element type: carbons (dark grey), hydrogens (light grey), nitrogen (blue) and oxygen (red). The molecular Δ$V_{\text{xc}}^{\text{Mol}}$ energy is given by a single thick turquoise line. Generic atom labels are used in the legend and correspond to Figure 2 in the main text.

**Figure S12.** Relative Δ$E_{\text{intra}}^{\text{A}}$ total atomic energies for the scan across ψ for Gly (top), Val (middle) and Ile (bottom). Only the highest energetically fluctuating atoms are plotted. Element colours are used for plot lines to distinguish element type: carbons (dark grey), hydrogens (light grey), nitrogen (blue) and oxygen (red). The molecular Δ$E_{\text{intra}}^{\text{A}}$ energy is given by a single thick dark orange line. Generic atom labels are used in the legend and correspond to Figure 2 in the main text.

**Figure S13.** Relative Δ$V_{\text{cl}}^{\text{AA'}}$^’^ total atomic energies for the scan across ψ for Gly (top), Val (middle) and Ile (bottom). Only the highest energetically fluctuating atoms are plotted. Element colours are used for plot lines to distinguish element type: carbons (dark grey), hydrogens (light grey), nitrogen (blue) and oxygen (red). The molecular Δ$V_{\text{cl}}^{\text{AA'}}$ energy is given by a single thick dark purple line. Generic atom labels are used in the legend and correspond to Figure 2 in the main text.

**Figure S14.** Relative Δ$V_{\text{xc}}^{\text{AA'}}$^’^ total atomic energies for the scan across ψ for Gly (top), Val (middle) and Ile (bottom). Only the highest energetically fluctuating atoms are plotted. Element colours are used for plot lines to distinguish element type: carbons (dark grey), hydrogens (light grey), nitrogen (blue) and oxygen (red). The molecular Δ$V_{\text{xc}}^{\text{Mol}}$ energy is given by a single thick turquoise line. Generic atom labels are used in the legend and correspond to Figure 2 in the main text.

**Figure S15.** Alteration of Figure 5c, with the addition of a further summed Δ$V_{\text{cl}}^{\text{Mol}}$ (purple) + Δ$E_{\text{intra}}^{\text{Mol}}$ (orange) energy (represented as brown). Energies are in kJmol^-1^ for the Ile ϕ scan.
